# Male-Specific Alleviation of Iron-Induced Striatal Injury by Inhibition of Autophagy

**DOI:** 10.1371/journal.pone.0131224

**Published:** 2015-07-06

**Authors:** Li-Fang Wang, Kazunari K. Yokoyama, Tzu-Yin Chen, Hsiu-Wen Hsiao, Pei-Chi Chiang, Ya-Ching Hsieh, Steven Lo, Chin Hsu

**Affiliations:** 1 Department of Medicinal and Applied Chemistry, College of Life Science, Kaohsiung Medical University, Kaohsiung, Taiwan; 2 Graduate Institute of Medicine, Kaohsiung Medical University, Kaohsiung, Taiwan; 3 Department of Physiology, Faculty of Medicine, College of Medicine; Kaohsiung Medical University, Kaohsiung, Taiwan; 4 Department of Medical Research, E-Da Hospital, I-Shou University, Kaohsiung, Taiwan; 5 Canniesburn Plastic Surgery Unit, Royal Infirmary, Glasgow, United States of America; University of Michigan, UNITED STATES

## Abstract

Men exhibit a worse survival rate than premenopausal women after intracerebral hemorrhage (ICH), however, no sex-specific management has been concerned. In a rat model involving infusion of ferrous citrate (FC) that simulates iron accumulation after hemorrhage, a higher degree of autophagy associated with higher injury severity was observed in striatum of males than in females. Since the imbalance between the levels of autophagy and energy demand may lead to cell death, we proposed that FC-induced autophagy is detrimental in a male specific manner and autophagy modulation affects injury severity in a sex-dependent manner. Rapamycin, an autophagy inducer, and conditional knockout gene of autophagy-related protein 7 (*Atg7*) in dopamine receptor D2 (DRD2) neurons were used to test our hypothesis using a mouse model with striatal FC infusion. The result showed that the levels of autophagic cell death and injury severity were higher in male than in female mice. Pre-treatment of FC-infused females with rapamycin increased the FC-induced behavioral deficit and DRD2 neuron death. However, DRD2 neuron-specific knockout of *Atg7* decreased FC-induced injury severity and the number of TUNEL(+) DRD2 neurons in males. These results suggest that autophagy in FC-infusion males is overactive with maladaptive consequences and inhibition of autophagy decreases the severity of FC-induced striatal injury in males. These findings present prospects for male-specific therapeutic strategy that targets autophagy in patients suffering from iron overload.

## Introduction

Primary intracerebral hemorrhage (ICH) results in the highest mortality of all stroke subtypes and less than 40% of ICH survivors regain their independence [1]. The incidence of ICH is higher among man, and their survival rate is lower compared with that of premenopausal women [2, 3]. While current treatments for stroke are primarily limited to supportive management [4, 5]. Previous reports indicated that men exhibit worse free radical homeostasis and weaker defense capacities against oxidative brain damage than women [6]. Our previous study also revealed that the levels of neuroprotective thioredoxin in the caudate nucleus of male rats were less than those in females after FC infusion and thioredoxin mediates the neuroprotective effect of estradiol in females but not in males [7]. Recent reports indicates that sex specific differences exist in both constitutive autophagy [8] and autophagy in the brain following hypoxia-ischemia [9]. These results imply a sexual dimorphism in the mechanism by which neurons respond to stress or to neuroprotectant. However, no sex-specific therapies have been considered for patients suffering from neurodegeneration following hemorrhage or iron intoxication.

Following ICH, accumulation of ferrous iron causes oxidative damage and contributes to the long-term neurological deficits [10, 11] and striatum is the area where ICH common occurred [12]. Oxidative stress and organelle damage induce autophagy that acts as an essential recycling mechanism for survival [13]. However, over-activation of autophagy has been recognized in conditions of severe oxidative stress following hypoxic/ischemic brain injury [14]. Previous results showed that striatal infusion of FC, which simulates iron accumulation after ICH, causes more autophagy and greater injury severity in male rats than in females [15]. Whether autophagy plays a sex-specific detrimental role in striatal injury caused by iron overload is unknown. In the present study, we infused FC into striatum of mice to mimic ferrous iron accumulation after ICH and used conditional knockout of *Atg7* to address the issue whether autophagy inhibition is beneficial for patients suffering from neurodegeneration caused by iron overload in a male-specific manner.

## Materials and Methods

### Intact and castrated mice

A total of 132 age-matched (12-week-old) [16] male and female C57BL/6 mice (National Laboratory Animal Center, Taipei, Taiwan) were used to examine the sex difference in FC-induced autophagy and the effect of rapamycin on FC-induced striatal injury. No mice were excluded. Mice implanted with estradiol (E_2_) after castration were used to study the contribution of E_2_ to the sex difference in FC-induced autophagy and injury in striatum. For the female mice, ovariectomy involved a small incision in both flanks after which the ovaries were removed. In the male mice, the skin and body wall caudal to the penis were incised with a scissor, and the testicles were retracted through the incision. The exposed testicular artery and vein were double-ligated and cut.[17] A Silastic tube (2 mm inner diameter, 20 mm in length) containing 0.38 mM E_2_ (Sigma-Aldrich, E8515) was implanted subcutaneously 24 h before FC infusion.

### Mice deficient for *Atg7* in DRD2 neurons

The *Atg7*-conditional knockout mice (*Atg7* ^*F/F*^) (a gift from Dr. Masaaki Komatsu, Laboratory of Frontier Science, Tokyo Metropolitan Institute of Medical Science, Bunkyo-ku, Tokyo, Japan) were cross-bred with transgenic mice expressing *Cre* recombinase under the control of the *Drd2* promoter (*Drd2-Cre*) (BAC-CRE drd2-44, Mutant Mouse Regional Resource Centers) to produce mice deficient for *Atg7* in the DRD2 neurons, which are abundant in the striatum [18]. The F1 heterozygote male (n = 20) and female (n = 20) littermates (*Atg7* ^+/–^ *Drd2-Cre*) were used. Equal number of *Atg7* ^*F/F*^ age-, weight- and sex-matched mice was used as control. To avoid the dilution by heterozygote knockout and by *Atg7* in other non-DRD2 cells, immunohistochemical detection, which is more sensitive than Western blot analysis, was used to check the knockout efficiency.

### Rapamycin pre-treatment and FC infusion

In mouse striatum, the caudate nucleus and the putamen are not distinguishable. Therefore, three microliters of rapamycin (50 nmol/L; LC Laboratories, Woburn, USA)[19], was infused into the right striatum (coordinates: 0.2 mm anterior, 2.5 mm lateral, and 3.5 mm ventral to bregma) using a microinfusion pump (CMA Microdialysis, Kista, Sweden) at a rate of 1 μL/min [15]. Five hours after rapamycin-pre-treatment [15], 3 μL of FC (1 mmol/L) (Sigma-Aldrich, St. Louis, USA) was infused into the right striatum at the same stereotaxic coordinates as the rapamycin injection. Two days later, the forelimb use asymmetry test was performed to evaluate FC-induced functional deficits. Then, mice were sacrificed and the brain tissue containing striatum was sampled for histological examination and immunihistochemical analysis. The samples for Western blot were dissected at 2 mm anterior and 2 mm posterior of the injection site, then separated the outer cortex and isolated the striatum. The iron overload was confirmed by Prussian blue assay on brain sections from mice infused with FC as shown in **S1 Fig**.

### Forelimb use asymmetry test

Individual mice were placed in a transparent cylinder in the dark, and the use of the ipsilateral limbs (I), contralateral limbs (C), and simultaneous use of both forelimbs (B) were observed for a 5-min period. The test was randomized, blinded, and repeated twice for each mouse. The forelimb use asymmetry score = I/(I+C+B)-C/(I+C+B) [20].

All of the above animal manipulations were approved by the Kaohsiung Medical University Committee for the Use of Experimental Animals. The IACUC approval numbers are 101001; 101106; and 102117.

### Western blot analysis

An equal amount of protein from each sample was separated by SDS-PAGE, transferred onto a PVDF membrane (NEN Life Science Products, Massachusetts, USA), recognized by rabbit anti-LC3 (microtubule-associated protein light chain 3) antibody (1:2000) (Sigma), rabbit anti-α-II spectrin antibody (1:1000, Santa Cruz), or mouse anti-β-actin antibody (1:5000) (Chemicon, CA, USA), followed by secondary antibody (goat anti-rabbit 1:5000 or goat anti-mouse 1:10,000); then visualized by ECL chemiluminescence (Perkin Elmer, Massachusetts, USA). The relative level of LC3-II to LC3-I depicted the level of autophagy [21]. The ratio of spectrin breakdown products with molecular weights of 145 or 150 kDa (SBDP 145/150) to spectrin served as an index of injury severity [22].

### Histological lesion

The paraffin-embedded tissues were serially sectioned into 10-μm thick slices. After hematoxylin and eosin (HE) staining, the extents of the histological lesions in every fifteenth section of the striatum were analyzed using Image-proPlus software (Universal Imaging Corp., Pennsylvania, USA), according to the staining intensity. The ratio of ipsilateral hemispheric volume of the striatum to the contralateral hemispheric volume served as an index of histological lesion [15].

### Immunostainning and TUNEL staining

Mouse anti-DRD2 antibody (1:100; Santa Cruz) was used for the identification of the DRD2-containing neurons. FITC-conjugated AffiniPure goat anti-rabbit IgG (1:200, Jackson ImmunoResearch Laboratories, West Grove, USA) and rhodamin-conjugated AffiniPure goat anti-mouse IgG (1:200, Jackson) were used to recognize rabbit anti-LC3B antibody (1:50; Sigma) and mouse anti- beclin-1(BECN1) antibody (1:50; BD), respectively. DAPI (Sigma) was used for nuclear labeling. Terminal deoxynucleotidyl transferase-mediated nick end labeling (TUNEL) solution containing FITC-dUTP (Roche, Basel Schweiz, Switzerland) was used for the detection of DNA fragmentation. Autophagic cell death was identified by the presence of TUNEL (+) BECN1 immunoreactive cells with intact nuclei [23]. The fields selected for quantification are around the lesion site resided in striatum as shown in panel (C) of **S2 Fig**, because the cells in the injection site are scarce as shown in the panel (B) of **S2 Fig**. Five fields per section and night sections per animal were selected for quantification.

### Automated quantification of TUNEL (+) DRD2 immunoreactive cells

After TUNEL staining and DRD2 immunostaining, the images of the entire striatum were scanned using a TissueFAXS, followed by *in situ* quantification using microscopy-based multicolor tissue cytometry (MMTC). Then, TissueQuest analysis software (TissueGnostics) was used to analyze TUNEL (+) DRD2 immunoreactive cells. The fluorescence intensities of the negative control samples defined the levels of unspecific staining and allowed us to determine the individual cut-off value for each sample.

### Statistical analyses

FC-induced injury severity was compared between brains of males and females using two-way ANOVA, followed by Scheffé *post hoc* test. Data related to the effects of rapamycin and *Atg7* knockout on FC-induced injury severity, autophagy, and DNA fragmentation were analyzed using multi-way ANOVA. Significance was accepted at the level of p<0.05.

## Results

### Levels of FC-induced autophagy, autophagic cell death and injury severity in the striatum of male and female mice

**Fig 1:**
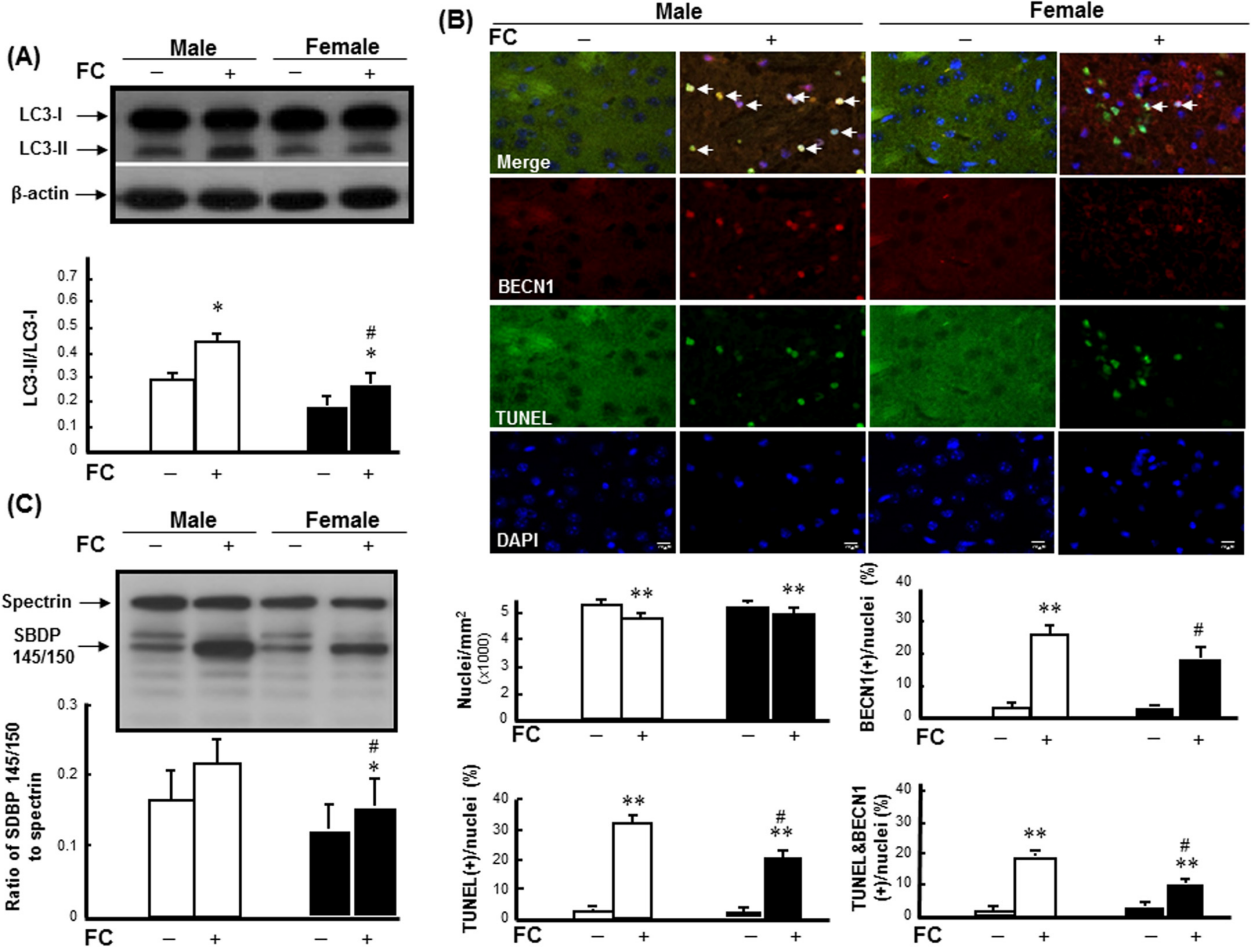
FC induced higher levels of autophagy, autophagic cell death, and injury severity in males. **(A) FC-induced LC3 lipidation.** White column: male group; black column: female group. **(B) Upper panel: Representative images of TUNEL(+) BECN1-immunoreactive cells.** The yellow spots indicated by arrows depicted TUNEL(+) BECN1-immunoreactive cells with round (intact) nuclei, which were regarded as indicators of autophagic cell death. **Lower panel: Numbers of DAPI-positive nuclei, TUNEL(+) nuclei, BECN1(+) cells, and TUNEL(+) BECN1-immunoreactive cells.** The number of immunoreactive cells was counted in every fifteenth section of the striatum under fluorescent microscope. Five fields in each tissue section were randomly selected for counting. Male: white column; female: black column. Data are presented as the means ± SDs (6 mice in each group). **(C) FC-induced injury severity.** The ratio of SBDP 145/150 to spectrin depicts injury severity. Data are presented as the means ± SDs (n = 6), *p<0.05, **p<0.01 compared with sex-matched saline-infusion controls; #p<0.05 compared with the FC-infusion males.

To examine the sex differences in FC-induced autophagy and injury severity, the levels of LC3-II, autophagic cell death and SBDP 145/150 were detected in striatum of C57BL/6 mice after FC infusion. The results revealed that FC infusion caused increases of LC3-II (Fig 1A) and autophagic cell death, which is simultaneously TUNEL positive and BECN1 immunoreactive (Fig 1B), in male and female mice. After quantification, the numbers of nuclei in the striatums of both males and females were decreased following FC infusion (Fig 1). Additionally, FC infusion significantly increased the percentages of TUNEL(+) nuclei, BECN1(+) cells, and TUNEL(+) BECN1 immunoreactive cells by 32%, 27%, and 19% in males, respectively, and 22%, 19%, and 10% in females, respectively (Fig 1B). Moreover, FC infusion caused increases of 33% and 24% in the ratios of SBDP 145/150 to spectrin (Fig 1C) in male and female mice, respectively.

### Role of E_2_ in sex differences in FC-induced autophagy and injury severity

**Fig 2:**
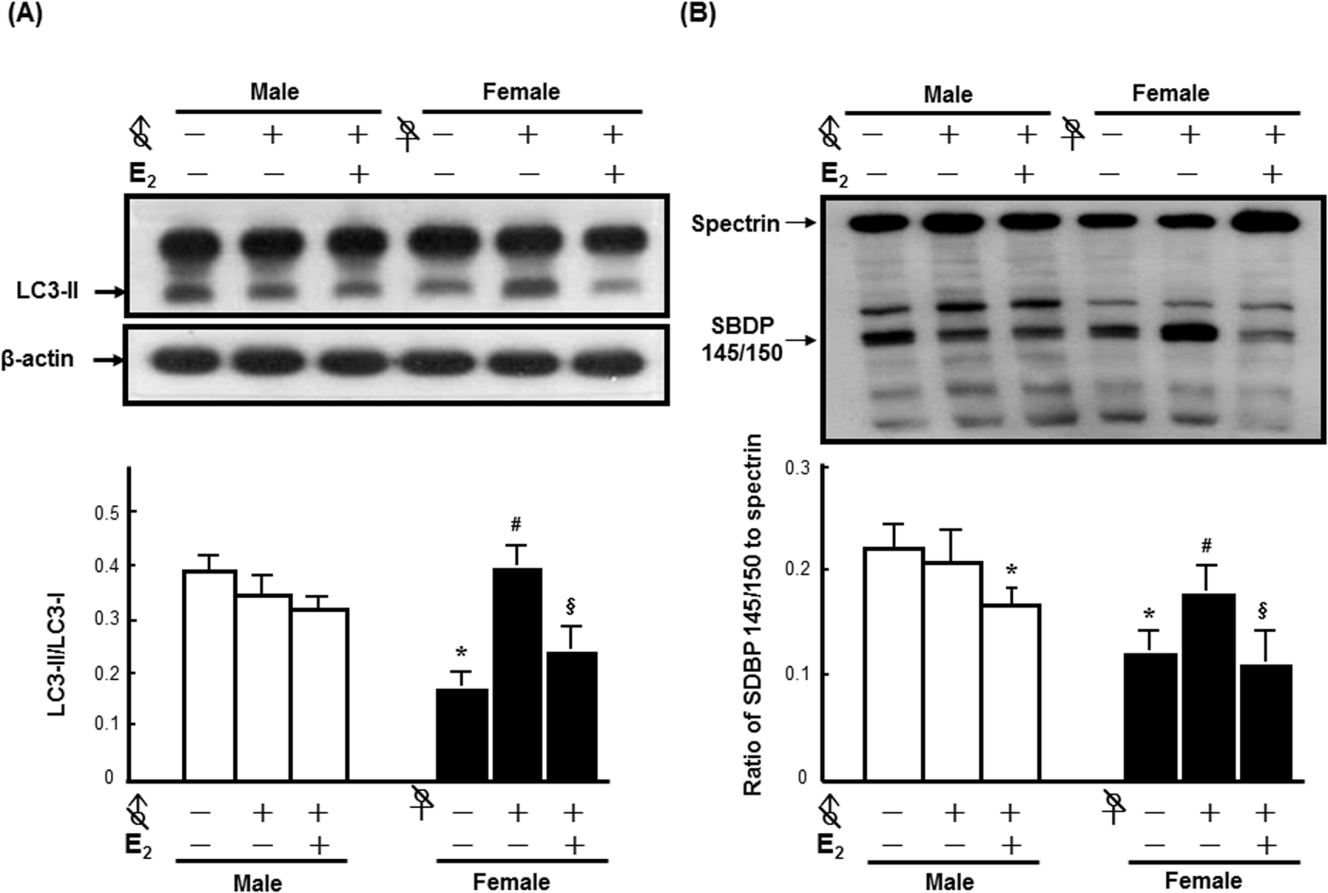
E_2_ contributed to the sex differences in FC-induced autophagy and injury severity in the striatum. All of the mice in every groups were infused with 3 μL of FC (1 mmol/L) into the right striatum. **(A) E_**2**_ reversed the increase of FC-induced LC3 lipidation by ovariectomy. (B) E_**2**_ decreased the ratios of SBDP 145/150 to spectrin in both sterilized female (♀ with a slash) and male (♂ with a slash) mice after FC infusion.** The data are expressed as the means ± SDs (n = 6), *p<0.05 compared with intact males; #p<0.05 compared with intact females; §p<0.05 compared with ovariectomized females.

To determine whether E_2_ contributed to the sex differences in FC-induced autophagy and injury severity in the striatum, both male and female C57BL/6 mice were sterilized prior to the implantation of E_2_ to exclude the influence of endogenous sex hormones. The results revealed that no significant effect of orchidectomy on FC-induced LC3 lipidation was observed, while ovariectomy increased the levels of FC-induced LC3 lipidation by 1.57-fold. Although E_2_ implantation slightly, but non-significantly, decreased the levels of FC-induced LC3 lipidation in the castrated male mice by 11%, the level of LC3-II in E_2_ implanted castrated males was lower than that of intact males by 25%. Moreover, E_2_ decreased FC-induced LC3 lipidation in the ovariectomized females by 39%, whereas no significant difference of the level of LC3-II between E_2_ implanted ovariectomized females and intact females was observed (Fig 2A). No significant effect of orchidectomy on FC-induced injury severity was observed in the male group, while ovariectomy markedly increased the FC-induced injury severity. E_2_ implantation decreased the ratios of SBDP 145/150 to spectrin in both sterilized male and female mice by 13% and 42%, respectively (Fig 2B).

### Female-specific effects of rapamycin on FC-induced injury severity and DNA fragmentation in DRD2 neurons

**Fig 3:**
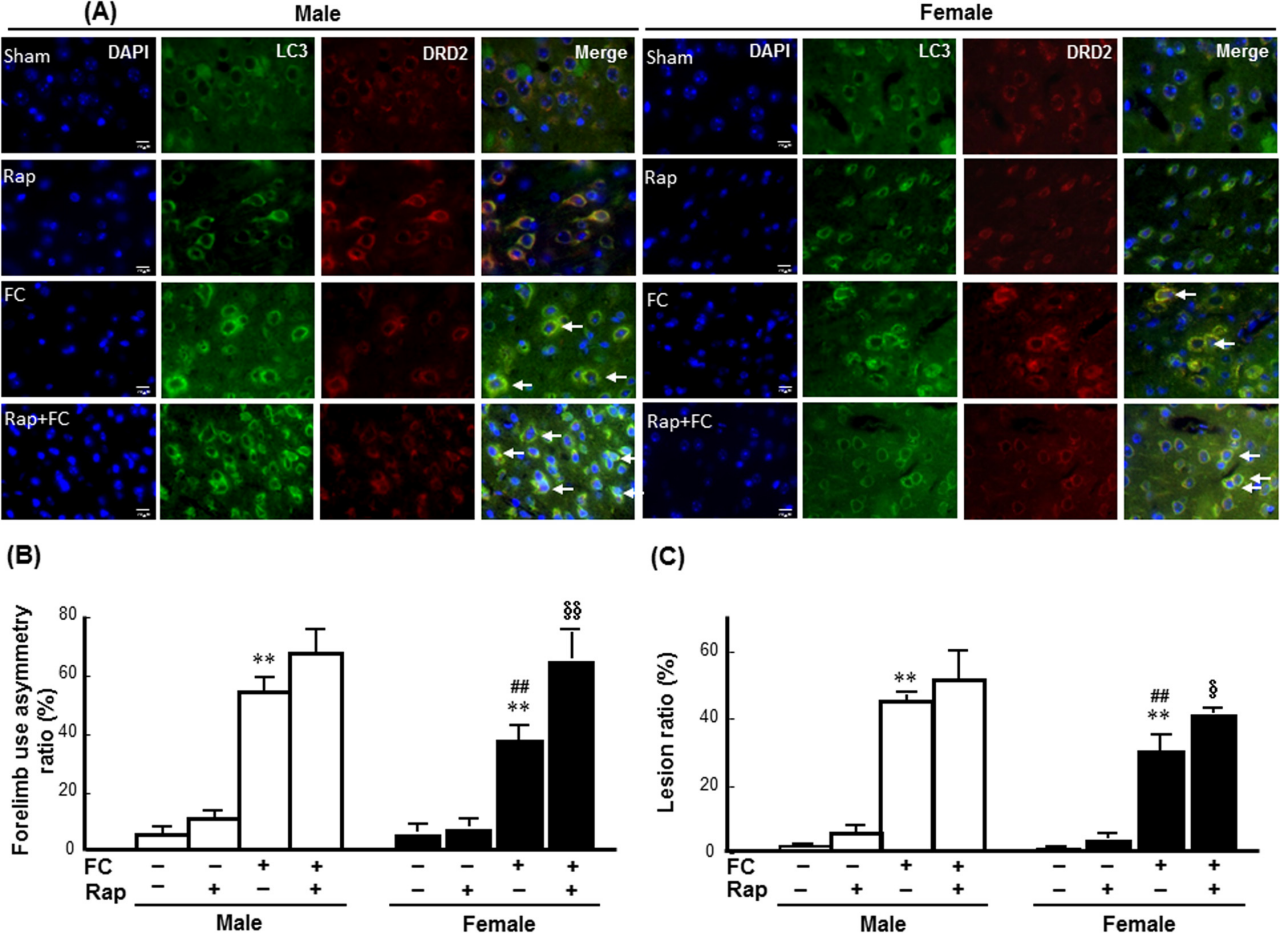
Rapamycin exaggerated FC-induced striatal injury in female mice. **(A) Rapamycin (Rap) enhanced the LC3 immunoreactivity in DRD2 neurons of both male and female striatum.** The pictures were captured under fluorescent microscope after immunostaining. Arrows indicated the merged nuclei surrounded by LC3 and DRD2 immunoreactivity depicted DRD2 neurons with autophagic features. **(B) Rapamycin exaggerated FC-induced behavioral deficits in the female group. (C) Rapamycin increased the histological lesion ratio caused by FC infusion in the females.** The data are expressed as the means ± SDs (n = 6). **p<0.01 compared with sex-matched saline-infusion control. ##p<0.01 compared with the FC-infusion males. §p<0.05, §§p<0.01 compared with the FC-infusion females.

To examine whether autophagy induction affected FC-induced striatal injury in a sex-dependent manner, the effects of rapamycin on FC-induced injury, behavioral deficits, histological lesion, and the numbers of TUNEL(+) DRD2 neurons were compared between the intact male and female C57BL/6 mice. As shown in Fig 3A, immunoreactivity of LC3 was co-localized with the DRD2 neurons in both male and female mice with or without FC infusion. The histological changes after FC infusion and the images of brain sections after HE stain were showed in the **S4 Fig**. The quantitative result showed that rapamycin increased both the FC-induced forelimb use asymmetry scores and the lesion ratios by 63% (Fig 3B) and 33% (Fig 3C), respectively, in female mice but did not affect these measurements in the males.

**Fig 4:**
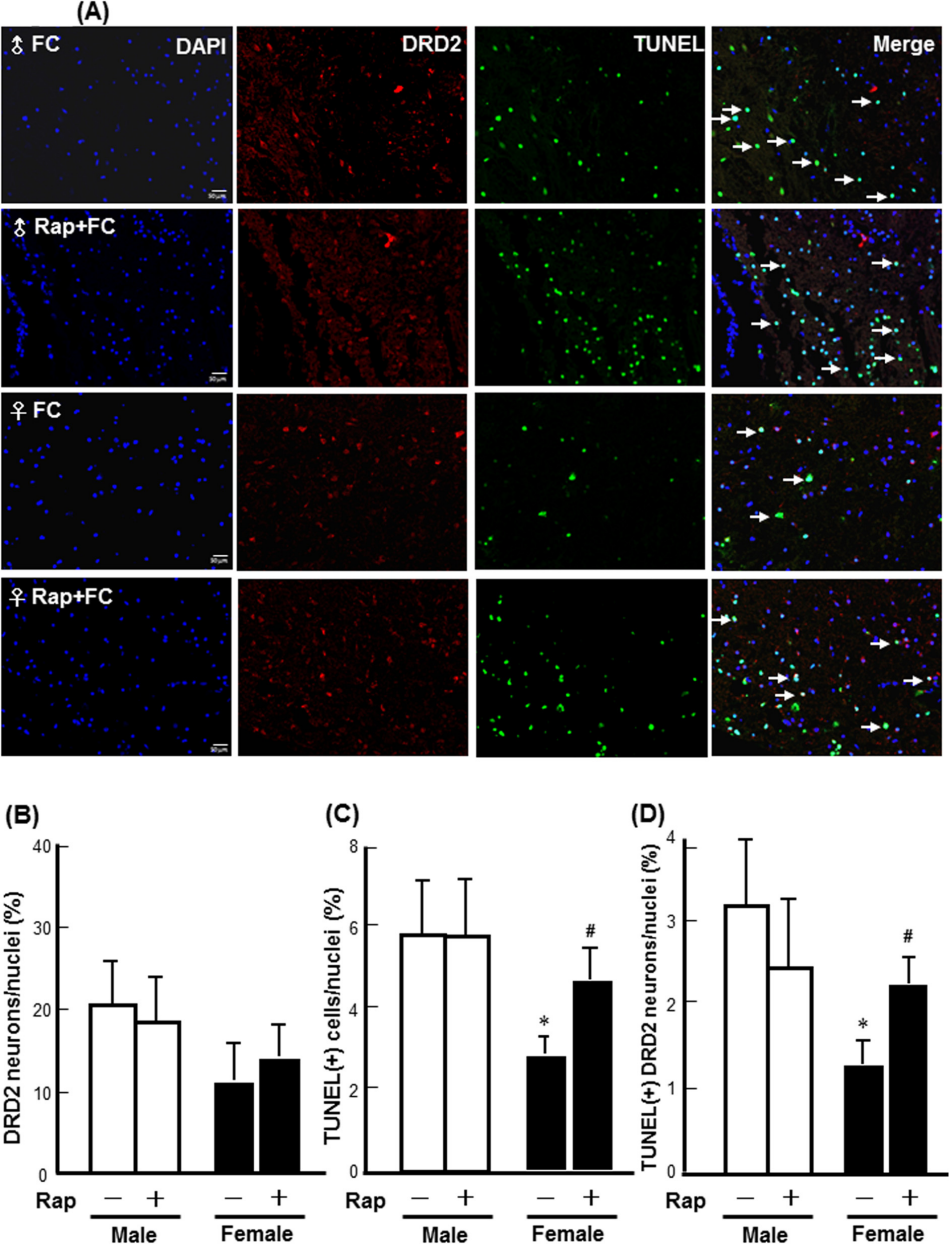
Rapamycin increased FC-induced DNA fragmentation in the DRD2 neurons of female mice. **(A) Representative images of TUNEL(+) DRD2 immunoreactive cells.** The images were captured using TissueFAXS. The merged bright yellow dots depict DRD2 neurons with DNA fragmentation, as indicated by the arrows. **(B) DRD2 neurons in total cells. (C) TUNEL(+) cells in total nuclei. (D) TUNEL(+) DRD2 neurons in total nuclei.** The tissue sections were scanned using a TissueFax cytometer, and the number of TUNEL(+) and/or DRD2 immunoreactive neurons were analyzed by TissueQuest. The data are expressed as the means ± SDs (n = 6). *p<0.05 compared with FC-infusion male; #p<0.05 compared with female mice without rapamycin pre-treatment.

To examine whether the increase in autophagic activity correlates with cell death in neurons, we examined LC3 staining along with neutron marker (NeuN) staining, while the result showed that LC3 immunoreactivity exhibited in both neuron and glial cells and the LC3 antibody reacted with both LC3-I and LC3-II **(S2 Fig)**. Moreover, TUNEL staining along with neutron marker (NeuN) or astrocyte marker (GFAP) staining were performed. The result showed that DNA fragmentation also exhibited in both neuron and astrocyte **(S3 Fig)**. Therefore, we quantified the number of TUNEL positive DRD2 neuron that is more correlative to behavioral deficit due to striatal injury using automated TissueFAXS followed by TissueQuest analysis. The results revealed that FC infusion increased the numbers of TUNEL(+) DRD2 neurons in the striatums of both the male and female mice (Fig 4A). Rapamycin did not change the percentages of DRD2 neurons in the total nuclei of either male or female mice (Fig 4B). Whereas, rapamycin increased the percentages of TUNEL(+) cells and TUNEL(+) DRD2 neurons by 63% and 73%, respectively, in the striatum of the FC infusion female mice but not in males following FC infusion (Fig 4C & 4D).

### Male-specific effects of *Atg7* knockout (KO) in DRD2 neurons on FC-induced injury severity and DNA fragmentation in DRD2 neurons

**Fig 5:**
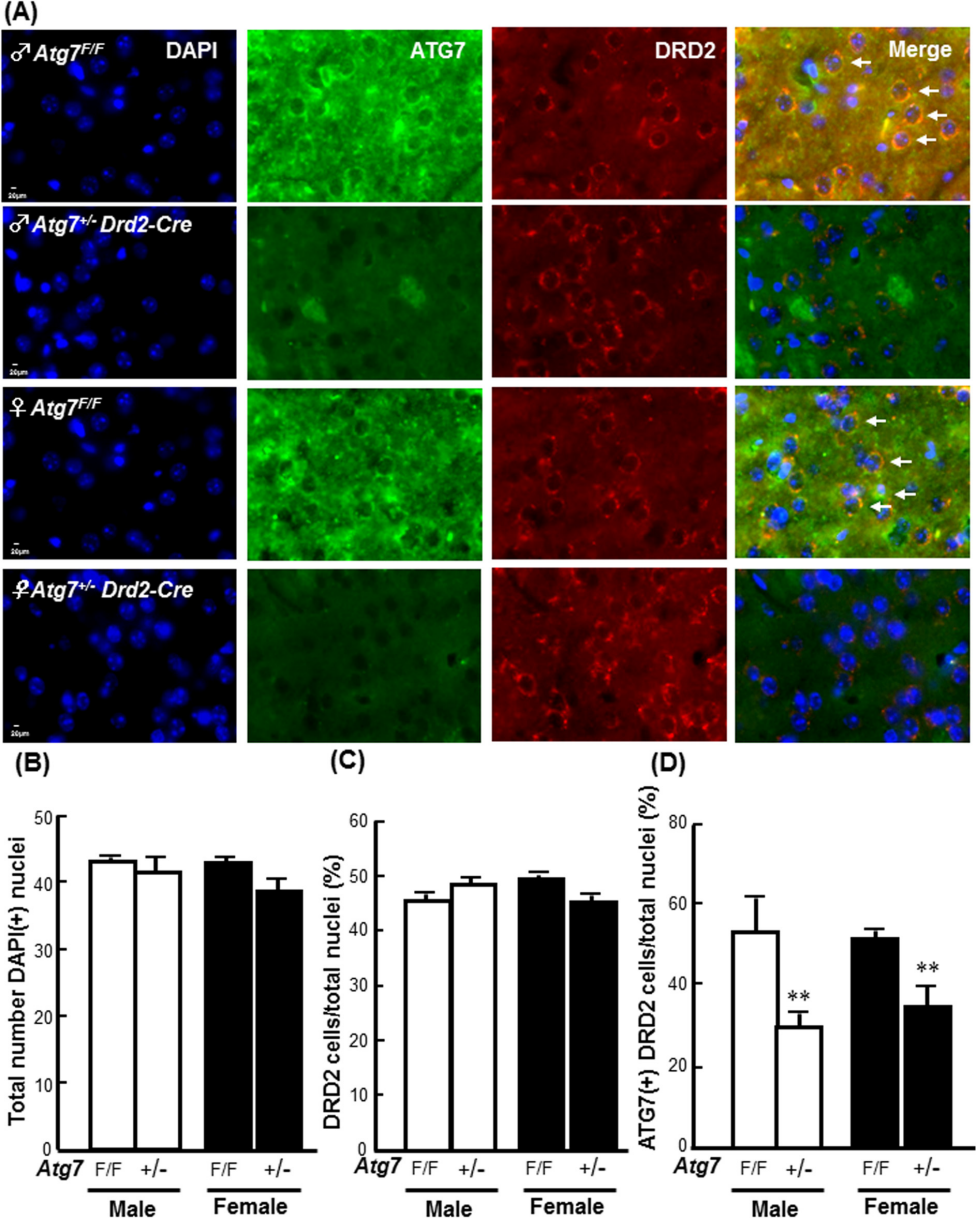
Heterozygous *Atg7* knockout in striatal DRD2 neurons. **(A) Representative images of ATG7 immunoreactive DRD2 neurons.** The pictures were captured under fluorescent microscope after immunostaining. Arrows indicate blue nuclei surrounded by immunoreactivities of ATG7 and DRD2. **(B) total number of DAPI(+) nuclei. (C) percentage of DRD2 neurons in total nuclei. (D) percentage of ATG7 immunoreactive DRD2 neurons in total nuclei.** The number of immunoreactive cells was counted in every fifteenth section of the striatum. Five fields in each tissue section were randomly selected for counting. Data are presented as the means ± SDs (number of mice was 4 in each group). *Atg7 KO* significantly decreased the percentage of ATG7(+) DRD2 neurons in the striatum of both male (white column) and female (black column) mice. **p< 0.01 compared with sex-matched *Atg7* ^*F/F*^ groups.

To address the effects of neuron-specific autophagy inhibition on iron toxicity, heterozygote-conditional knockout mice in which *Atg7* was deleted in the DRD2 neurons (*Atg7* ^*+/-*^ *Drd2-Cre*) were used. The knockdown efficiency of *Atg7* in the DRD2 neurons was confirmed by counting the numbers of ATG7-immunoreactive DRD2 neurons. As shown in Fig 5A, in both the male and female groups, the numbers of ATG7-immunoreactive DRD2 neurons were decreased in the striatums of the *Atg7* ^*+/-*^ *Drd2-Cre* mice compared with those of the *Atg7* ^*F/F*^ mice. The representative images of striatum containing brain sections after HE stain were showed in the **S5 Fig**. The quantitative result showed that there were no differences in the total numbers of nuclei or the percentages of DRD2 neurons in the total nuclei between the *Atg7* ^*+/-*^ *Drd2-Cre* and wild-type *Atg7* ^*F/F*^ mice (Fig 5B and 5C). The percentages of ATG7-immunoreactive DRD2 neurons in the striatums of the male and female *Atg7* ^*+/-*^ *Drd2-Cre* mice were significantly lower than those in the male and female *Atg7* ^*F/F*^ mice by 44% and 33%, respectively (Fig 5D).

**Fig 6:**
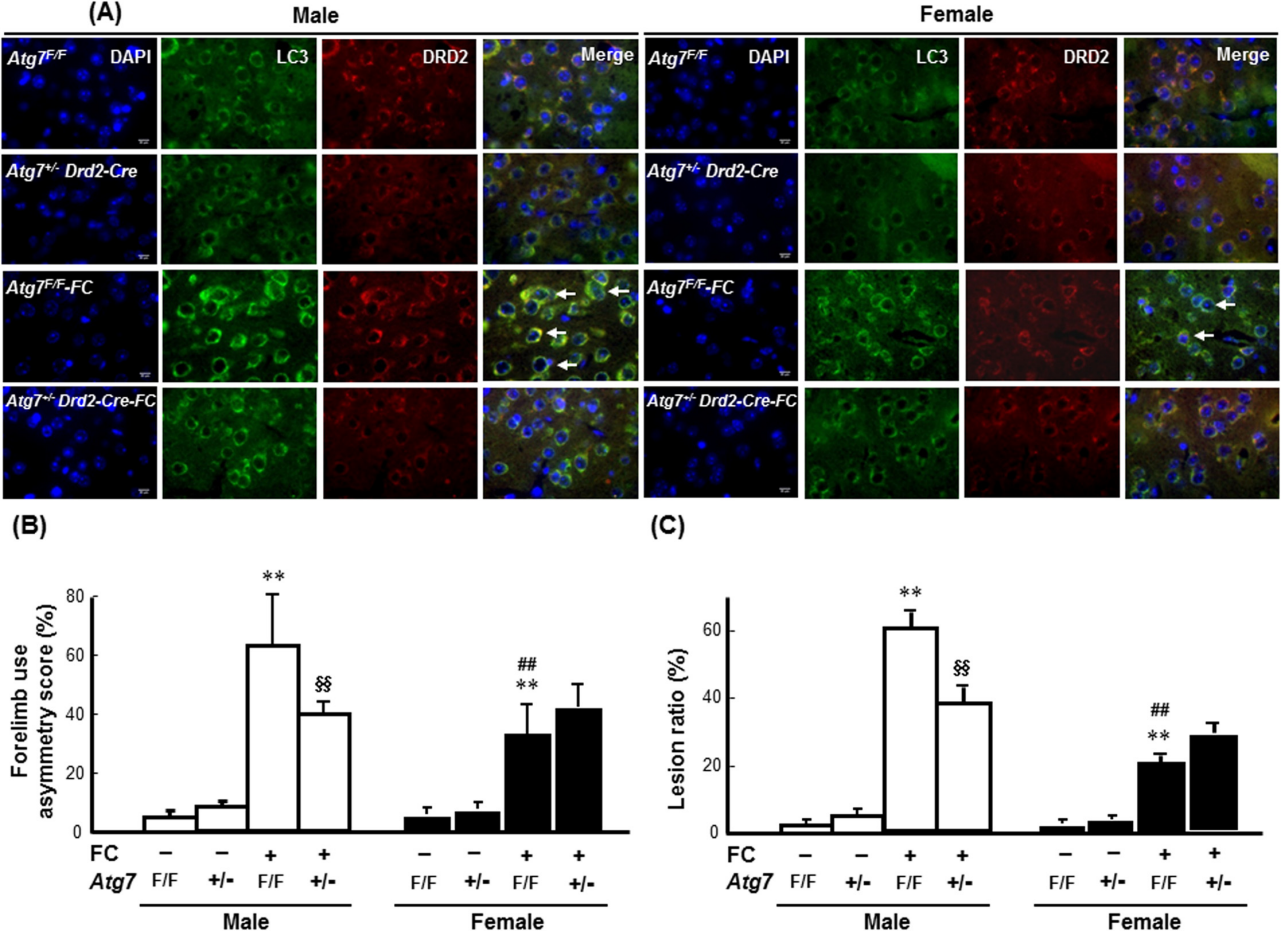
Knockout of *Atg7* in DRD2 neurons decreased FC-induced striatal injury in male and female mice. **(A) *Atg7* KO decreased LC3 immunoreactivity in the DRD2 neurons.** The images were captured using TissueFAXS. Arrows indicate LC3 immunoreactive DRD2 neurons. **(B) *Atg7* KO decreased FC-induced behavioral deficits in males but not in females. (C) *Atg7* KO decreased the lesion ratios caused by FC infusion in males only.** The data are expressed as the means ± SDs (n = 6). **p<0.01 compared with the sex-matched *Atg7* ^*F/F*^ groups; ##p<0.01 compared with the FC-infused *Atg7* ^*F/F*^ male mice. §§p<0.01 compared with the FC-infused *Atg7* ^*F/F*^ male mice.

Both the male and female *Atg7* ^*+/-*^ *Drd2-Cre* mice exhibited lower levels of FC-induced LC3 aggregation in the striatum compared with the *Atg7* ^*F/F*^ mice (Fig 6A). In males, *Atg7* knockout decreased both FC-induced behavioral deficits (Fig 6B) and histological lesions (Fig 6C) by 36% (p<0.01) and 36% (p<0.01), respectively. By contrast, in females, no significant effect of *Atg7* KO on the FC-induced behavioral deficits and histological lesions was observed (Fig 6B and 6C).

**Fig 7:**
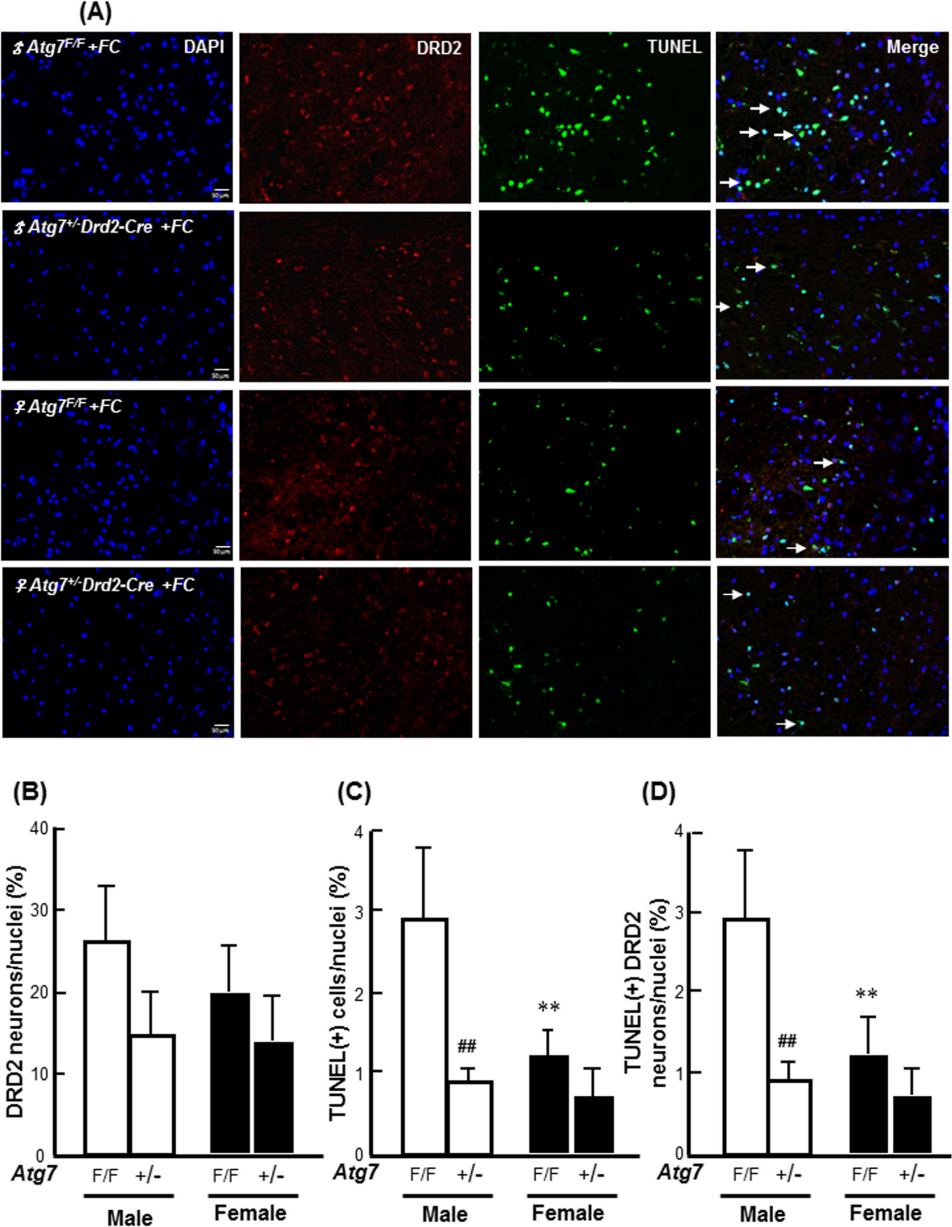
*Atg7* KO mice exhibited a male-specific benefit in FC-induced DNA fragmentation in the DRD2 neurons in the striatum. **(A) Representative images of the TUNEL(+) DRD2 neurons.** The bright green-yellow dots depict DRD2 neurons with DNA fragmentation, as indicated by the arrows. Quantitative results of the percentages of DRD2 neurons **(B), TUNEL(+) cells (C), and TUNEL(+) DRD2 neurons (D).** The data are expressed as the means ± SDs (n = 6). **p<0.01, *Atg7* ^*F/F*^ female vs. *Atg7* ^*F/F*^ male; ##p<0.01 *Atg7* ^*F/F*^ vs. *Atg7* ^*+/-*^ *Drd2-Cre*.

Moreover, the results from TissueQuest analysis showed that no significant sex differences in the numbers of DRD2 neurons were found between the *Atg7* ^*F/F*^ and *Atg7* ^*+/-*^ *Drd2-Cre* mice (Fig 7A & 7B). FC infusion caused a significantly higher level of DNA fragmentation in male than in the female *Atg7* ^*F/F*^ mice. Interestingly, in males, *Atg7* knockout decreased the percentages of TUNEL(+) cells and TUNEL(+) DRD2 neurons by 70% and 69%, respectively, following FC infusion. By contrast, no significant differences in the percentages of TUNEL(+) cells or TUNEL(+) DRD2 neurons between the *Atg7* ^*+/-*^ *Drd2-Cre* and *Atg7* ^*F/F*^ females following FC infusion were observed (Fig 7C and 7D).

## Discussion

The present study demonstrated that the striatum of male mice were more susceptible to FC-infusion that simulates iron accumulation after ICH. Enhancement of autophagy exaggerated the FC-induced striatal injuries and behavioral deficits in females but not males, and inhibition of neuronal autophagy decreased FC-induced striatal injuries and behavioral deficits in males but not in females. These results suggest that the over-activation of autophagy plays a harmful response to striatal iron overload in males but not in females. For the first time, this study explored the sex-dimorphic role of autophagy induction in striatal injury caused by iron overload.

Men are more vulnerable to ICH [24] and exhibit worse survival rates than premenopausal women following ICH [3]. Previously, a rat models of ICH with autologous whole blood infusion into basal ganglia, both brain edema and behavioral deficits were more severe in males than in females [25]. Recently, a rat model involving infusion with ferrous iron, which contributes to the neurological deficit following ICH, into the caudate nucleus showed that males are more vulnerable to iron overload [7, 15]. Presently, a mouse model of striatal FC infusion shows a higher level of autophagy associated with a higher injury severity in males than in females, implicating a possible harmful role of FC-induced autophagy in males. Additionally, ovariectomy, which simulates menopause in women, increased FC-induced striatal autophagy and injury severity, while E_2_ implantation decreased FC-induced autophagy and injury severity in the ovariectomized mice. These results supported the supposition that E_2_ contributes to the lower level of FC-induced autophagy and injury severity in females. Lacking of the protection conferred by endogenous estrogen may explain their vulnerability to FC-infusion in males and ovariectomized-females.

After ICH, degradation of hemoglobin releases ferrous iron, which undergoes a redox cycle such as Haber-Weiss reaction and/or a Fenton reaction in the presence of citric acid, causes persistent conversion of oxygen into reactive oxygen species that trigger cell death. In addition to apoptosis and necrosis, which contribute to the ICH/FC-induced neuronal death [26] [27], ROS generated by ferrous ions also triggers autophagy and/or autophagic cell death [28, 29]. The induction of autophagy, a process for organelle turnover and bulk protein degradation [13], has been reported in patients with critical illnesses [30] and in the rat model of ICH [31]. The beneficial or harmful role of autophagy under stress might be determined by the balance/imbalance between the levels of autophagy and energy demand. In the present result, rapamycin pre-treatment slightly but not significantly increased the FC-induced autophagic cell death and injury severity in males. It is possible that the combination of rapamycin pre-treatment and FC-infusion reached the plateau of autophagy induction. Because, autophagy is an ATP-dependent process and the energetic dysfunction existed under oxidative stress due to iron overload. Interestingly, a prominent increase of autophagic cell death and injury severity was observed in FC infusion females pre-treated with rapamycin compared with FC infusion females without rapamycin pre-treatment. It suggests that pre-treatment with rapamycin diminishes the limiting effect of female endogenous E_2_ on FC-induced autophagy, which in turn becomes harmful au1tophagic cell death. E_2_ has been reported to interact with ERα in plasma membrane that is in a complex with ER-interacting protein, which facilitates estrogen activation of PI3K/Akt. Since mTOR (autophagy inhibitor) is a downstream target of PI3K/Akt [32], rapamycin (a mTOR inhibitor) may interfere E_2_ signaling via affecting mTOR. However, the other possible mechanism underlying the effect of rapamycin on interfering E_2_ signaling may not be excluded due to the non-specific effects of rapamycin/mTOR on downstream signaling. It actually remains unknown what factors determine whether autophagy is beneficial or pathogenic. An improved understanding of neuronal autophagy will provide novel insights into the pathogenic mechanisms of dysfunctional autophagy that underlie neurodegeneration due to iron overload and, ultimately, help develop therapeutic interventions.

On the other hand, conditional knockout of *Atg7* in DRD2 neurons decreased the percentage of TUNEL(+) DRD2 neurons and diminished FC-induced injury severity in males, while slightly increased the FC-induced injury severity in females. These results strongly suggested that, in males, the inhibition of FC-induced autophagy could switch a maladaptive or harmful autophagy to a response in favor of neuroprotection. Whereas, in females, the moderate level of FC-induced autophagy might be sufficient for supplying energy demand, thus inhibition of autophagy deprived the protective machinery under oxidative stress. Accordingly, male-specific inhibition of autophagy might be a useful therapeutic strategy for patients with iron overload.

In conclusion, over-activation of the autophagic machinery plays a maladaptive and harmful role in FC-induced striatal injury in males but not in females. Inhibition of autophagy could represent a useful therapeutic target for iron-induced brain injury in men or postmenopausal women who have lost the limitation of autophagy by estrogen. Therefore, this study presents new paths for exploring sex-specific therapeutic strategies that address the maladaptive over-activation of autophagy, such as gene therapy targeting autophagy-related genes.

## Supporting Information

S1 Fig The Prussian blue assay on brain section from mice infused with or without ferrous citrate (FC). Two days after FC infusion, the brain tissue containing striatum was sectioned and stained by working solution which is a mixture of equal parts of hydrochloric acid and potassium ferrocyanide prepared immediately before use. Arrows in the lower left panel indicate dark blue deposits that confirmed the iron overload after FC infusion.
(TIF)

S2 Fig Ferrous citrate (FC)-induced autophagy in both neuron and astrocyte around the lesion site, but not in the lesion site within the striatum. (A) Striatum without FC infusion; (B) Lesion site; (C) Around lesion site. Bal/c mice were stereotaxically infused with 3 μL FC into the right striatum. Two days after FC infusion, the brain tissue containing striatum was sectioned and stained by LC3 antibody for LC3-II aggregation (green dots) as an autophagic marker; NeuN for neuron marker; and DAPI for nuclei. Bright green spots (arrow) showed in the lower raw of right panel indicate LC3 aggregation, which are exhibited in both neuron and non-neuronal cells.
(TIF)

S3 Fig Ferrous citrate (FC) induced DNA fragmentation in both neuron and astrocyte. Bal/c mice were stereotaxically infused with 3 μL FC into the right striatum. Two days after FC infusion, the brain tissue containing striatum was sectioned and stained by LC3 Ab (Green), in the left panel; TUNEL (Green) and GFAP Ab (Red) in the left panel; and TUNEL (Green) and NeuN Ab (Red) in the right panel. DAPI (Blue) was used to identify nucleus simultaneously. Bright green spots showed in the left panel indicate LC3 aggregation, which is a detection marker of autophagy. Arrow showed in the lower raw of right panel indicates the TUNEL positive astrocyte; arrowhead showed in the lower raw of right panel indicates the TUNEL positive neuron.
(TIF)

S4 Fig Rapamycin pre-treatment exaggerated the histological lesion in striatum of females after FC infusion. The paraffin-embedded tissues were serially sectioned into 10-μm thick slices. After hematoxylin and eosin (HE) staining, the extents of the histological lesions were analyzed using Image-proPlus according to the staining intensity in the enclosed area. The ratio of ipsilateral hemispheric volume of the striatum to the contralateral hemispheric volume served as an index of histological lesion. The images at the upper panel indicate the higher magnifications the histological injury is more obvious. The scale bars represent the different magnifications under microscope.
(TIF)

S5 Fig *Atg7* knockout diminished the histological lesion in striatum of males after FC infusion. The paraffin-embedded tissues were serially sectioned into 10-μm thick slices. After hematoxylin and eosin (HE) staining, the extents of the histological lesions in every fifteenth section of the striatum were analyzed using Image-proPlus software, according to the staining intensity in striatum. The ratio of ipsilateral hemispheric volume of the striatum to the contralateral hemispheric volume served as an index of histological lesion.
(TIF)
