# Supplementary figures and images for: Male-Specific Alleviation of Iron-Induced Striatal Injury by Inhibition of Autophagy
Source: PLoS One. 2015 Jul 6;10(7):e0131224. doi: 10.1371/journal.pone.0131224 (PMC4492841; doi:10.1371/journal.pone.0131224)

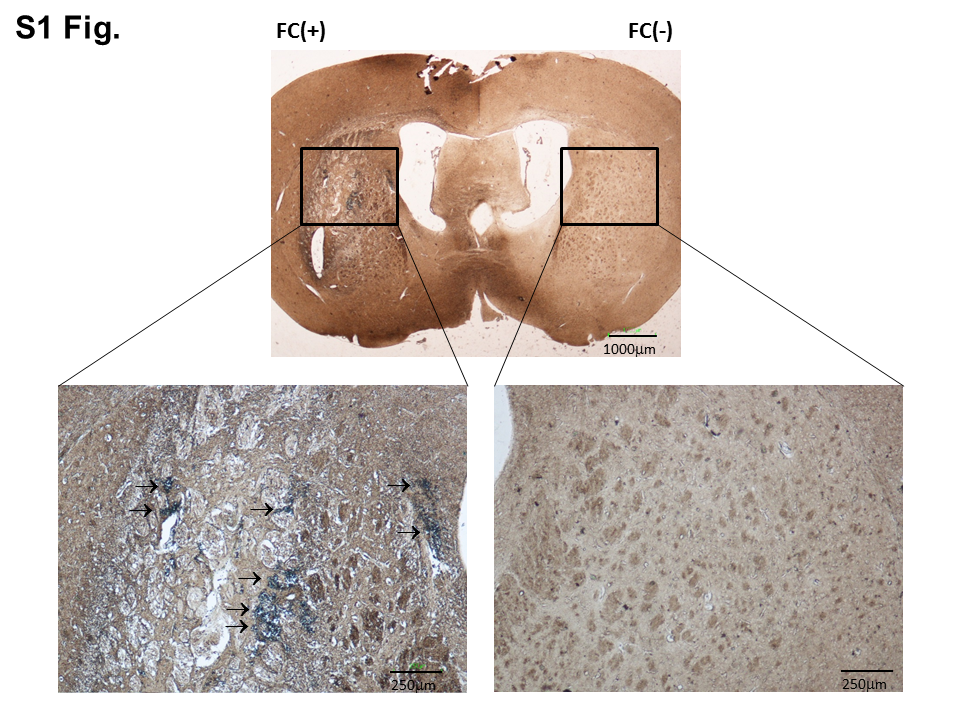

Supplement: S1 Fig — Two days after FC infusion, the brain tissue containing striatum was sectioned and stained by working solution which is a mixture of equal parts of hydrochloric acid and potassium ferrocyanide prepared immediately before use. Arrows in the lower left panel indicate dark blue deposits that confirmed the iron overload after FC infusion. (TIF) [file pone.0131224.s001.tif]

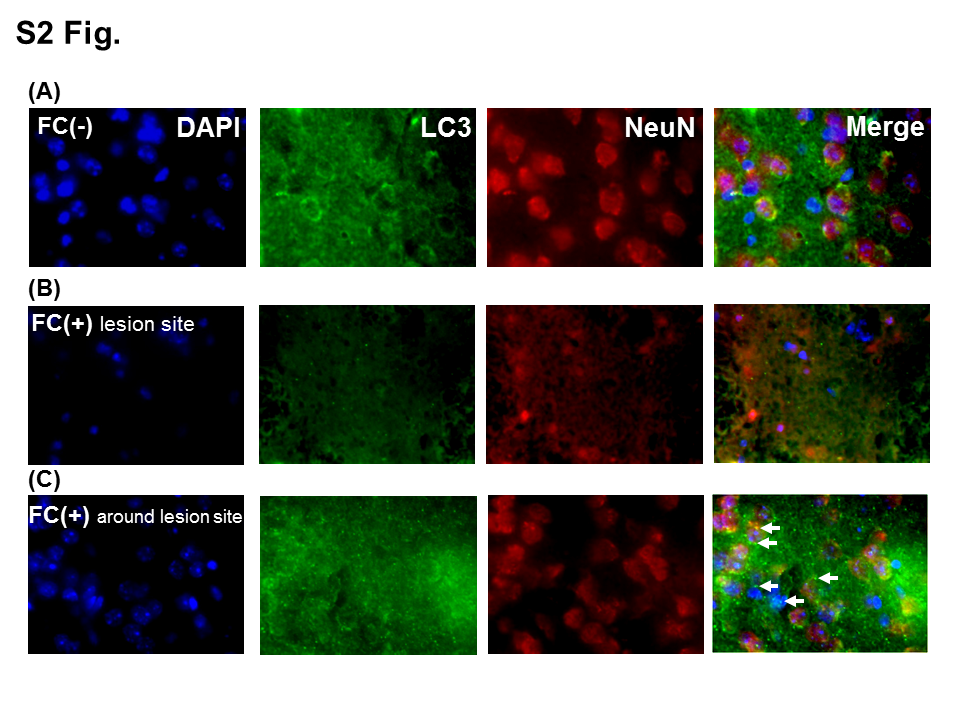

Supplement: S2 Fig — (A) Striatum without FC infusion; (B) Lesion site; (C) Around lesion site. Bal/c mice were stereotaxically infused with 3 μL FC into the right striatum. Two days after FC infusion, the brain tissue containing striatum was sectioned and stained by LC3 antibody for LC3-II aggregation (green dots) as an autophagic marker; NeuN for neuron marker; and DAPI for nuclei. Bright green spots (arrow) showed in the lower raw of right panel indicate LC3 aggregation, which are exhibited in both neuron and non-neuronal cells. (TIF) [file pone.0131224.s002.tif]

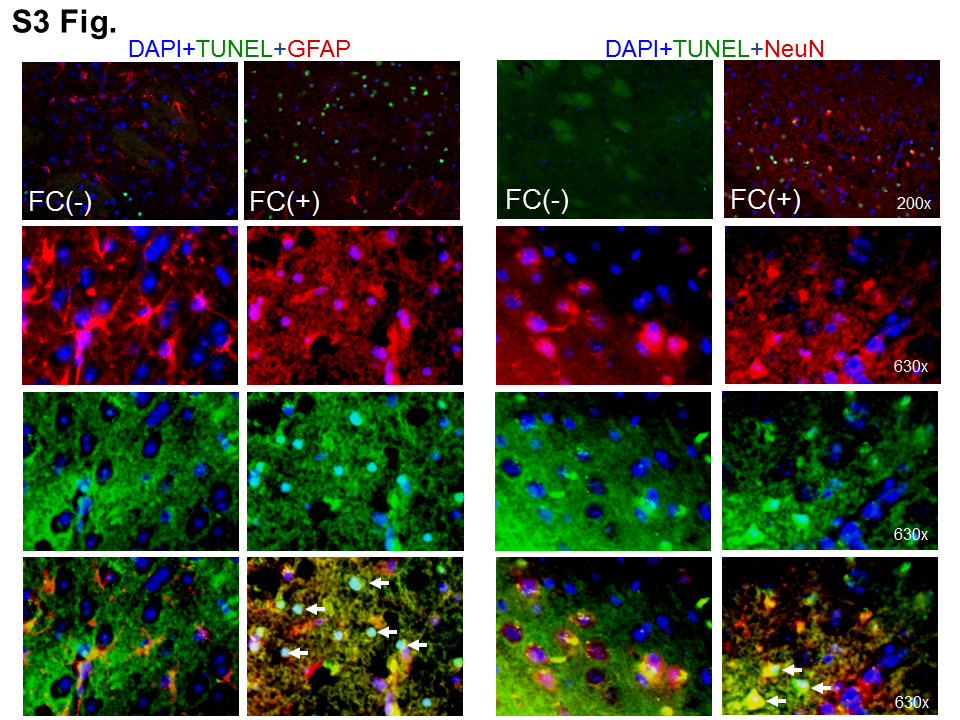

Supplement: S3 Fig — Bal/c mice were stereotaxically infused with 3 μL FC into the right striatum. Two days after FC infusion, the brain tissue containing striatum was sectioned and stained by LC3 Ab (Green), in the left panel; TUNEL (Green) and GFAP Ab (Red) in the left panel; and TUNEL (Green) and NeuN Ab (Red) in the right panel. DAPI (Blue) was used to identify nucleus simultaneously. Bright green spots showed in the left panel indicate LC3 aggregation, which is a detection marker of autophagy. Arrow showed in the lower raw of right panel indicates the TUNEL positive astrocyte; arrowhead showed in the lower raw of right panel indicates the TUNEL positive neuron. (TIF) [file pone.0131224.s003.tif]

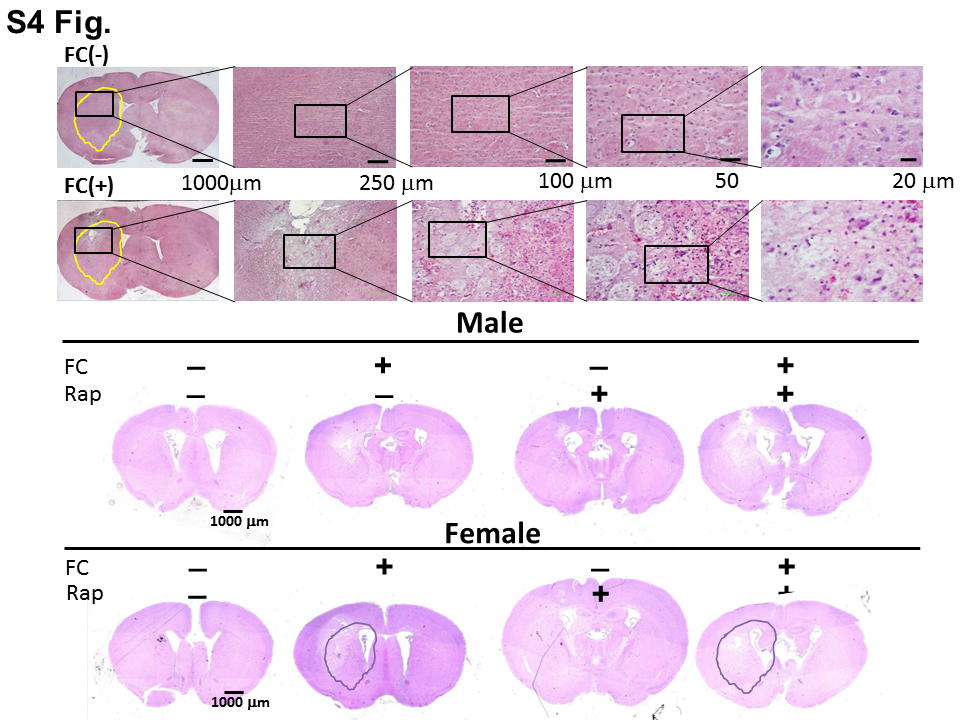

Supplement: S4 Fig — The paraffin-embedded tissues were serially sectioned into 10-μm thick slices. After hematoxylin and eosin (HE) staining, the extents of the histological lesions were analyzed using Image-proPlus according to the staining intensity in the enclosed area. The ratio of ipsilateral hemispheric volume of the striatum to the contralateral hemispheric volume served as an index of histological lesion. The images at the upper panel indicate the higher magnifications the histological injury is more obvious. The scale bars represent the different magnifications under microscope. (TIF) [file pone.0131224.s004.tif]

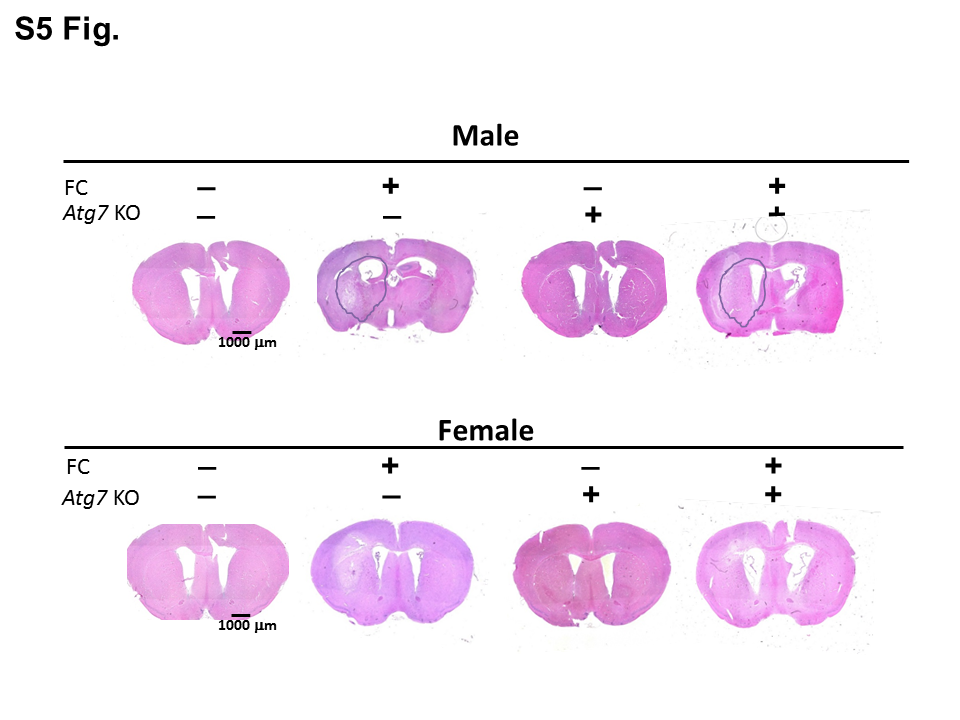

Supplement: S5 Fig — The paraffin-embedded tissues were serially sectioned into 10-μm thick slices. After hematoxylin and eosin (HE) staining, the extents of the histological lesions in every fifteenth section of the striatum were analyzed using Image-proPlus software, according to the staining intensity in striatum. The ratio of ipsilateral hemispheric volume of the striatum to the contralateral hemispheric volume served as an index of histological lesion. (TIF) [file pone.0131224.s005.tif]
